# Progress report on multiple endocrine neoplasia type 1

**DOI:** 10.1007/s10689-025-00440-4

**Published:** 2025-01-18

**Authors:** Reut Halperin, Amit Tirosh

**Affiliations:** 1 Faculty of Medicine, Tel Aviv University, Tel Aviv, Israel; 2 Neuroendocrine Oncology Unit, Division of Endocrinology, Diabetes and Metabolism, Sheba Medical Center, Ramat Gan, Israel; 3 The Chaim Sheba Medical Center, ENTIRE – Endocrine Neoplasia Translational Research Center, Tel Aviv University Faculty of Medicine, 2 Sheba Road, Tel HaShomer, Ramat Gan, Israel

**Keywords:** MEN, Neuroendocrine tumors, Pituitary, Hyperparathyroidism

## Abstract

Multiple endocrine neoplasia type 1 (MEN1) syndrome is an autosomal dominant disorder caused by a germline pathogenic variant in the *MEN1* tumor suppressor gene. Patients with MEN1 have a high risk for primary hyperparathyroidism (PHPT) with a penetrance of nearly 100%, pituitary adenomas (PitAd) in 40% of patients, and neuroendocrine neoplasms (NEN) of the pancreas (40% of patients), duodenum, lung, and thymus. Increased MEN1-related mortality is mainly related to duodenal-pancreatic and thymic NEN. Management of PHPT differs from that of patients with sporadic disease, as the surgical approach in MEN1-related PHPT includes near-total or total parathyroidectomy because of multigland hyperplasia in most patients and the consequent high risk of recurrence. NEN management also differs from patients with sporadic disease due to multiple synchronous and metasynchronous neoplasms. In addition, the lifelong risk of developing NEN requires special considerations to avoid excessive surgeries and to minimize damage to the patient’s function and well-being. This progress report will outline current insights into surveillance and management of the major clinical manifestation of MEN1 syndrome in children and adults with MEN1 diagnosis. In addition, we will discuss MEN1-like clinical presentation with negative *MEN1*-genetic workup and future clinical and research directions.

## Introduction

Multiple endocrine neoplasia type 1 syndrome (MEN1, OMIM #131100) is a rare autosomal dominant disorder. It is caused by a germline loss of function pathogenic variant (PV) in the *MEN1* gene located on chromosome 11q13 [1]. The main clinical manifestations are the “three P’s”: early-onset primary hyperparathyroidism (PHPT) with multiple gland involvement, pituitary adenoma (PitAd), mostly prolactinomas or non-functioning PitAd, and neuroendocrine neoplasms (NEN) mainly pancreatic (PNEN) but also duodenal, bronchial and thymic NEN [2, 3], as detailed in Table 1. Additional manifestations include adrenocortical adenoma (rarely – adrenocortical carcinoma), meningioma, and ependymoma, as well as various skin manifestations (angiofibromas, lipomas, collagenomas) [4, 5]. Recent studies suggested a possible increased risk of other malignancies, including thyroid and breast neoplasms [6], with current French MEN1 guidelines recommending breast screening for women with MEN1 [7].

**Table 1:** Clinical manifestations of multiple endocrine neoplasia type 1

Manifestation			Penetrance by age 80	Age of diagnosis Years Median [range]	Comments
**PHPT**			95%	31 [10–65]	Usually hyperplasia involving all parathyroid glands
**PitAd**			30-46%	28 [14–60]	PRL-secreting 54–62% NF PitAd 14–31% GH-secreting 8% PRL + GH-co-secreting 2–9% ACTH-secreting 4%
**NEN**	Duodeno-Pancreatic		90% by age 70	41 [13–73]	Multiple secretory phenotypes Gastrinoma (ZES) 54% NFPNEN 18–68% Insulinoma 18%
	Gastric		13–23% (of those with ZES)		Almost exclusively in the presence of ZES
	Lung		17.6%	43 [18–66]	
	Thymic		7.3%	39–47	
**Adrenal**	Adenomas, ACC, PHEO		23–73% (age not reported)	47 [NA]	
**Skin**	Lipomas 17–33% Angiofibromas 22–88% Collagenomas 0–72% Melanoma 4–11%				
**Other**	Leiomeyoma < 10%				
	Meningiomas Ependymoma				Scarce data, no strong correlation to MEN1
	Breast cancer				Scarce data, no strong correlation to MEN1

ACC – adrenocortical carcinoma; ACTH – adrenocorticotroph hormone; GH-growth hormone; NF – non-functioning; PHEO – pheochromocytoma; PHPT – primary hyperparathyroidism; PitAd – pituitary adenoma; PRL- prolactin; NEN – neuroendocrine neoplasia; ZES – Zollinger-Ellison syndrome

Data are based on [5, 7–9, 26, 72, 106–110]

Overall penetrance is 98% by age 80, with 95% of the patients developing PHPT, 41% PNEN, and 30% PitAd [7, 8]. The median age at diagnosis is 31 years (range 10–65) for PHPT, 41 (13–73) for DP-NEN, and 28 (14–60) for PitAd. In a study of up to four generations of patients with MEN1, the age at diagnosis was significantly younger in consecutive generations for PHPT, PitAd, PNEN, and lung NEN. These results suggest genetic anticipation or improved surveillance modalities [9]. Mortality is MEN1-related in 70% of cases, primarily due to thymic and duodenopancreatic NEN (DP-NEN) [10]. The term “age at diagnosis” for a specific manifestation in MEN1 is usually the time of diagnosis as part of the surveillance program. It is infrequent that PHPT will be diagnosed due to symptoms, and most PHPT diagnoses are based on biochemical workup. NEN can be diagnosed via imaging surveillance, and also, although less often, by symptoms related to hypergastrinemia or unregulated insulin secretion (hypoglycemia). PitAd may present as a small non-functional lesion in MRI, but also by over secretion of prolactin, growth hormone, or adrenocorticotropic hormone (ACTH), and by hypopituitarism, most often as a change in menstruation pattern in women.

The *MEN1* gene consists of ten exons and encodes the menin protein. Menin activity is tissue-specific, as a tumor suppressor in endocrine tissues and a pro-oncogene in hematopoietic tissues [11, 12]. In MEN1, there is a loss of heterozygosity (LOH) and reduced menin levels at the endocrine tumor tissue [13]. Germline pathogenic variants are scattered throughout the *MEN1* gene, with no specific hot spot [14–16]. Although a relatively large percentage of the *MEN1* variants are located in exons 2 and 10, this possibly reflects the proportionally large size of these exons. In addition, exon 9 also harbors a large proportion of the variants due to a possible founder effect [17].

While there is no clear genotype-phenotype correlation in MEN1, a recent study of the French MEN1 registry revealed an association between large rearrangements and earlier age of both PHPT and PitAd disease onset compared to patients with truncating or non-truncating variants [18]. However, the age of PHPT presentation was 28 years of age (range 10 to 74), and for PitAd, 30 years of age (range 10 to 74) [17], which are both comparable to the ages reported in the literature for the general MEN1 population [9]. Thus, a possible association between age of onset and variant type is not currently established. The Italian MEN1 cohort did not find a correlation between variant type and location and PHPT and PitAd age of onset and rate, yet found a higher frequency of PNEN in patients with nonsense (73%) vs. frameshift and missense variants (52% and 54%, respectively) and of thoracic NEN in patients with splice-site (18%) vs. frameshift variants (6%) [16].

### Indications for genetic testing for MEN1

The decision to pursue genetic testing depends on the clinical scenario, age at diagnosis, and family history, as summarized in Table 2. If a patient has either a first-degree relative with MEN1 or has any two diagnoses out of the “three P’s” (PHPT, PitAd, DPNEN), the need for a genetic workup is straightforward. However, other germline endocrine syndromes can have overlapping clinical manifestations with MEN1 and should also be included (most often by next-generation sequencing [NGS], either targeted gene panel sequencing or exome sequencing), especially if other clinical manifestations suggesting a syndrome are present [18].

**Table 2:** Indications for genetic evaluation in patients with suspected MEN1 syndrome

Clinical manifestation	Indication	Specific genes of interest
**Family history**	1st degree relative with MEN1 diagnosis	*MEN1*, *CDKN1B*, also consider other* *CDK* genes
**At least two diagnoses of the following in an individual**	PHPT PitAd DPNEN	*MEN1*, *CDKN1B*, also consider other *CDK* genes
**PHPT**	Age at diagnosis < 30 (or 45) Multiple glands involved Recurrence Negative localization Parathyroid carcinoma Other syndromic clinical diagnosis Family history	*MEN1*, *CDKN1B*,* CDC73*,* GCM2*,* RET* Possibly also *CaSR*,* GNA11*,* AP2S1*
**PitAd**	Age at diagnosis < 18 Age at diagnosis < 30 plus Invasive macroadenoma Other syndromic clinical diagnosis Family history	*MEN1*, *CDKN1B*,* AIP*,* GPR101* duplication, *PRKAR1A*, also consider other *CDK* genes and *SDHx* genes#
**DPNEN**	Multiple DPNEN Gastrinoma Age at diagnosis < 50	*MEN1*, *CDKN1B*,* VHL*,* NF1*,* TSC1*,* TSC2*, also consider other *CDK* genes and *GCGR*
**Thymic NEN**	Family history Young age at diagnosis? Any thymic NEN?	*MEN1*, *CDKN1B*, also consider other *CDK* genes
**Lung NEN**	Family history Young age at diagnosis?	*MEN1*, *CDKN1B*, also consider other *CDK* genes

DP – duodenopancreatic; PHPT – primary hyperparathyroidism; PitAd– pituitary adenoma; NEN – neuroendocrine neoplasia; * other *CDK* genes - *CDKN1A*, *CDKN1B*, *CDKN1C*, *CDKN2A*,* CDKN2C*; # *SDHx* genes – *SDHA*,* SDHAF2*,* SDHB*,* SDHC*,* SDHD*

For patients with PHPT, genetic testing is recommended if age at diagnosis is younger than 30 years of age (in some studies, younger than 50 years of age), there is multiple gland involvement, recurrence, parathyroid carcinoma, negative localization of the involved glands or a family history of PHPT [2, 7, 18–21]. Patients with PitAd should undergo genetic evaluation in case of a positive family history, age at diagnosis under 18 years of age or under 30 years of age, and with invasive macroadenomas [22, 23]. Multiple PNENs, gastrinoma, or occurrence of DPNEN before age 50 should also warrant genetic evaluation [7, 24]. There are no clear indications for genetic evaluation in patients with isolated LNEN or thymic NEN. However, some 25% of thymic NEN cases are associated with MEN1, so evaluation should be considered [25]. In addition, patients diagnosed with LNEN or thymic NEN at a young age should probably undergo genetic evaluation.

### Screening and follow-up

Diagnosis of MEN1 is based on either having two major clinical manifestations (PHPT, PitAd, and/or NEN), one major clinical manifestation-and a first-degree relative with MEN1, or carrying a known *MEN1* PV [2]. There is a controversy regarding the age of genetic evaluation in first-degree family members of patients with a diagnosis of MEN1. Some guidelines suggest genetic evaluation starting at five years of age for the presence of the familial pathogenic variant regardless of current clinical presentation [26], while others suggest genetic evaluation at ten years of age unless there are early-onset clinical manifestations in first-degree family members [7]. These evaluation suggestions probably stem from the low rate of any MEN1-related manifestation before the age of five and ten years (3% and 14%, respectively) [27].

In patients with MEN1, the goal of screening is two-fold: (1) Early detection of neoplastic processes, mainly regarding NEN of the duodenum, pancreas, lungs, and pituitary adenomas; and (2) Identification of endocrine alterations that require intervention (in PHPT) or of those alterations that may stem from the neoplastic process (PitAd, functional DP-NEN). Early detection of DP-NEN and lung NEN is mainly achieved via imaging and rarely due to clinical syndromes such as hypoglycemia in insulinoma or recurrent/resistant peptic ulcer (Zollinger-Ellison syndrome, ZES) in gastrinoma. Although several biochemical biomarkers correlated with disease burden in patients with MEN1 [28], there is currently no accurate and validated biochemical marker for early detection and follow-up of non-functional DP-NEN in this population [29]. This is especially true considering the wide use of proton pump inhibitors in the general population, which increases chromogranin A and gastrin levels as a byproduct, precluding their use as markers. Biochemical follow-up is required mainly for assessing parathyroid and pituitary (over-) function status and, if possible, using gastrin levels to evaluate patients with clinically suspected ZES. Current Endocrine Society MEN1 guidelines [2] and French Endocrine guidelines are similar with respect to most screening recommendations and are presented in Table 3 [2, 7]. Of note, a recent consensus on pancreatic neuroendocrine tumors in MEN1 suggested postponing the age of biochemical evaluation to 16 years of age [26]. Interestingly, in a recent publication of real-life screening and follow-up practices of MEN1 patients, there was marked heterogeneity in the use of biomarkers between centers [30] emphasizing the need for uniform and up-to-date guidelines. Indeed, updated guidelines and a consensus statement are expected in the upcoming months.

**Table 3:** Asymptomatic patients with MEN1 screening recommendations according to current guidelines

Site	Guideline	Age to begin biochemical Screening	Biochemical test	Age to begin imaging screening	Imaging test
**Parathyroid**	Thakker et al. 2012	8	Blood calcium + PTH	None	None
	Goudet et al. 2024	10	Blood calcium ± PTH	None	None
**Pituitary**	Thakker et al. 2012	5	Prolactin, IGF-1	5	Pituitary MRI (every 3 years)
	Goudet et al. 2024	10	Prolactin, IGF-1	10	Pituitary MRI
**Pancreatic NEN**	Thakker et al. 2012	5	Glucose, insulin	< 10	MRI, CT, or EUS (annually)
		< 10	Pancreatic polypeptide, VIP, glucagon, chromogranin A		
		20	Gastrin		
	Goudet et al. 2024	12	Blood glucose	12	Abdominal MRI
**Thymic NEN**	Thakker et al. 2012	None		15	CT or MRI (1–2 years)
	Goudet et al. 2024	None		20 (family history)/ 25(men)/ 30(women)	Chest MRI
**Bronchial/lung NEN**	Thakker et al. 2012	None		15	CT or MRI (1–2 years)
	Goudet et al. 2024	None		30	Chest MRI
**Adrenal**	Thakker et al. 2012	None		< 10	MRI or CT (annually)
	Goudet et al. 2024	None		12	Abdominal MRI
**Breast**	Thakker et al. 2012	None		None	
	Goudet et al. 2024	None		40	Mammography

CT, computerized tomography; EUS, endoscopic ultrasound; IGF-1, insulin-like growth factor; MEN1, multiple endocrine neoplasia type 1; MRI, magnetic resonance imaging; NEN, neuroendocrine neoplasm; PTH, parathyroid hormone

## Main manifestations

### Primary hyperparathyroidism (PHPT)

PHPT is the most common manifestation in MEN1 patients, reaching a penetrance of almost 100%, with age at diagnosis as early as 10 years of age [9] but typically at an older age, as the median age at presentation is 31 years. Screening includes calcium level with or without PTH level (Table 3), and those that screen positive undergo a complete PHPT diagnostic workup according to PHPT guidelines in the general population [31]. PHPT in patients with MEN1 usually manifests with mild hypercalcemia, yet with common secondary insults such as hypercalciuria with nephrolithiasis and reduced bone density [32, 33]. The diagnosis and surgical intervention criteria for PHPT in MEN1 are similar to those for the general population. However, allocation of the involved parathyroid glands in MEN1 requires special consideration: Multiple parathyroid gland hyperplasia is common, with higher rates of ectopic gland location and parathyroid carcinoma compared with PHPT in the general population [32, 34, 35], with 20% chance of ectopic gland location in patients with MEN1 requiring reoperation for recurrent PHPT [36].

The first line of treatment for PHPT in MEN1 is surgical intervention. The classic surgical approach for patients with MEN1 dictates open bilateral exploration with either subtotal (ST, 3-3.5 glands) removal or total parathyroidectomy with partial gland auto-transplantation (TP/AT) [2, 7]. Other surgical approaches include unilateral clearance (side of surgery determined based on imaging findings) or a selective resection, as in non-MEN1 PHPT with intraoperative PTH measurement [7].

There is controversy regarding performing preoperative imaging for parathyroid localization in MEN1. The expected multigland hyperplasia questions the utility of imaging preoperatively, as the surgical approach is not expected to change regardless of imaging results [2], as detailed below. However, the possible ectopic location and supernumerary parathyroid glands that may occur in MEN1 suggest the usefulness of imaging. If performed, the recommended imaging modalities are neck ultrasound and 99Tc-MIBI scintigraphy. A recent retrospective study demonstrated some advantages of preoperative localization by 18 F-fluorocholine PET/CT with a 73% detection rate compared with 61% and 58% detection rates using 99Tc-MIBI scintigraphy and ultrasound, respectively [7, 37]. We recommend performing preoperative localization imaging, including a neck ultrasound and 99Tc-MIBI scintigraphy, for all patients with MEN1 before PHPT surgery. The rationale is to assess whether there are identifiable additional/ectopic parathyroid glands and if some of the glands appear less hyperplastic than others, enabling better surgical planning.

Regarding surgical outcomes, the recurrence rate of PHPT in MEN1 is as high as 53% after an average ten-year follow-up [36], compared with up to 5% recurrence in the general population after surgery [38]. Recurrence is 24% for ST and 13% for TP/AT, with 12% and 7% hypoparathyroidism rates for ST and TP/AT, respectively, and reoperation rates of 24% for ST and 7% for TP/AT [39]. There is limited data on the optimal treatment of PHPT recurrence after surgery. The reoperation surgical approach includes neck exploration, with the possible advantage of measuring intraoperative PTH levels to assess adequate endocrine control [36]. Another invasive option is ethanol ablation, with one study reporting reaching normocalcemia in 73% of patients for a mean duration of two years but with an 8% risk for hypoparathyroidism [40]. Non-interventional treatment includes oral calcimimetics, with two randomized controlled trials of patients with MEN1 and PHPT demonstrating a significant reduction in serum calcium level [41, 42] and another study showing 32% serum calcium normalization in patients with PHPT recurrence [43]. The threshold for surgical intervention, the choice of imaging modalities (if used at all), and the surgical approach varies between different centers and depends on local expertise. Further data are required to establish the optimal treatment approach for PHPT recurrence.

### Pancreatic-duodenal neuroendocrine tumors (DP-NEN)

This section will discuss the complex diagnosis, monitoring, and management of MEN1-related DP-NEN. Neuroendocrine neoplasms (NEN) are associated with an increased risk for mortality in patients with MEN1. The relatively rare thymic NEN shows the highest risk (hazard ratio [HR] of 4.64), followed by functional glucagon, somatostatin, and vasoactive intestinal peptide (VIP) secreting PNEN (HR 4.29), non-functional PNEN (HR 3.43) and gastrinomas (HR 1.89) [10].

Similar to our considerations in the clinic, we will divide the discussion in this section according to the functional status of the tumors: non-functional DP-NEN, gastrinoma, and (pancreatic) insulinoma. Within the non-functioning neoplasms, we will discuss small DP-NEN, adequate surveillance and management, and the various medical and surgical options and considerations in this unique scenario. We will not discuss the management of advanced DP-NEN, that are based on the guidelines and recommendations for the management of patients with advanced sporadic DP-NEN [44]. Additionally, we will not discuss NEN of the lung, stomach, and thymus, which are typical manifestations of MEN1, but are less frequent than PD-NEN.

The management of patients with functional neuroendocrine neoplasms is bi-dimensional and requires taking into account both the oncological implications of the tumor and the endocrine derangements.

The endocrine manifestations are unique for each hormone oversecretion-tumor: gastrin oversecretion, leading to Zollinger-Ellison syndrome (ZES), can be readily controlled using proton-pump inhibitors (PPIs) while insulin oversecretion leading to hypoglycemia episodes might be more challenging to control. A main difference in the management of functional vs. non-functional tumors is the use of debulking of functional tumors for controlling oversecretion via surgical, radionuclide, or interventional radiology, even if complete resection is not feasible. In patients harboring advanced non-functional DP-NEN, the mainstay approach is systemic therapy rather than debulking surgery [44].

#### Functioning MEN1-related DP-NEN

In addition to the characteristics of functional DP-NEN in general, their presentation in the context of MEN1 adds complexity as they are not radiologically distinguishable (insulinoma cannot be readily separated from non-functioning PNEN) and complicating the distinction further there are reports on multiple insulinomas [45] and even glucagonomas [46] in the context of MEN1. The unique characteristics of gastrinomas when detected in patients with MEN1 are well known and should be taken into consideration [47], the two main differences from sporadic gastrinomas being the pronounced multifocality of MEN1-related duodenal gastrinomas and the potential for synchronous pancreatic head gastrinomas and other functional- and non-functional PNEN, again complicating a possible surgical treatment.

The topic of synchronous primary PNEN, when trying to locate gastrin- or insulin-secreting PNEN, can be approached using the interventional radiology-based technique of selective arterial calcium injection (SACI). In this approach, the hepatic vein is catheterized and sampled for gastrin [48] or insulin [49] (in accordance with the suspected oversecretion syndrome), while the pancreas arteries are injected with calcium or secretin in ZES. If an increase in gastrin/insulin is detected, then the region suspected to be vascularized by the artery injected with calcium/secretin is the target for resection.

The diagnosis and management of insulinoma follow the current guidelines for sporadic disease. Once there is a biochemical validation for insulinoma, it should be located and resected to avoid additional hypoglycemic episodes. It is important to note, especially in the context of MEN1, that ^68^Ga-DOTA-based PET/CT has low sensitivity for localized insulinomas, and in MEN1, where multiple pancreatic lesions are typical, it may identify a non-functioning PNEN rather than the insulinoma [50]. If only one PNEN is located, then surgical resection is the optimal approach that leads to cure in most cases. However, if not, a case-by-case evaluation by an experienced multidisciplinary team is required to delineate the optimal management plan.

#### Small non-functioning MEN1-related P-NEN

It is widely accepted that MEN1-related small non-functioning DP-NEN can be safely followed. However, the threshold for surgery varies between groups and recommendations, with some suggesting a tumor diameter of 10 mm [10, 51] as a threshold for resection and others 20 mm [52] The accumulating data supports size-based risk stratification and management planning. Indeed, in an international consensus statement on the management of MEN1-related NEN, a cutoff of 20 mm for resection was suggested for non-functioning DP-NEN unless aggressive features on imaging or biopsy were detected or if aggressive NEN was reported in the family history [26].

#### DP-NEN surveillance – imaging and tissue sampling

In a large retrospective study of the DutchMEN Study Group, including 413 patients (3477 imaging studies) [53], MRI had higher sensitivity and specificity than CT for detecting and ruling out PNEN. The authors concluded that MRI is the preferred non-invasive imaging modality based on excellent diagnostic accuracy. The ability to avoid ionizing radiation by using MRI is an additional advantage in a population that requires numerous recurrent scans.

Lewis et al. evaluated the preoperative assessment of patients with MEN1 undergoing PNEN resection [54]. In this retrospective study, 51 patients underwent PNEN resection, 88% of whom underwent distal pancreatectomy. In terms of imaging, endoscopic ultrasound (EUS) had the highest sensitivity and positive predictive value compared with CT and single-photon emission computed tomography (SPECT). However, the role of EUS in DP-NEN diagnosis in the hereditary context is different from its role in patients with sporadic disease. In MEN1, there is a high pretest probability for NEN in general. Hence, radiologically typical lesions on MRI do not require EUS-guided sampling. Furthermore, EUS will probably detect small, clinically insignificant NENs, while clinically significant NENs will probably be identified by MRI. Therefore, using EUS is not justified for routine surveillance considering the risk of the procedure, and should only be reserved for the rare cases in which a biopsy is needed.

In the DutchMEN cohort [53], biopsying pancreatic lesions in patients with MEN1 proved to be inaccurate: of 34 pancreatic lesions biopsied, 24 were PNEN, but of an additional six lesions that were defined as normal, cyst or unrepresentative by FNA, all were eventually found to be PNENs. In addition, four lesions were defined as adenocarcinoma based on FNA, but two were eventually PNEN. Since small DP-NENs do not require any interventions, identifying them as NEN does not contribute as it will not change the management plan. Hence, the high sensitivity of EUS vs. MRI or CT is not a useful advantage in MEN1 clinical practice. EUS and tissue sampling of pancreatic lesions in patients with MEN1 should be reserved for lesions that seem atypical radiologically or aggressive in terms of change over time or other radiological parameters.

^68^Ga-DOTA-based PET/CT is a functional imaging that targets cells expressing somatostatin receptors (SSTR), typical for neuroendocrine neoplasms. The sensitivity of 68Ga-DOTA-based PET/CT is higher than anatomic imaging both in patients with sporadic [55–58] and MEN1-related DP-NEN [59–61]. Similar to the high sensitivity of EUS, it is not necessarily beneficial to DP-NENs that are smaller than the MRI detection threshold. Nevertheless, in patients with advanced DP-NEN, in pre-surgical preparation where accurate staging is required, and in patients that are candidates for radioligand therapy with 177Lu-DOTA peptides, 68Ga-DOTA-based PET/CT is the optimal imaging modality [62, 63].

#### DP-NEN– prophylactic intervention

The most studied medical intervention for patients with MEN1-related DP-NEN are somatostatin analogs, which are well established as a first-line intervention for patients with sporadic, well-differentiated gastric-enteropancreatic NEN [64, 65]. In MEN1, a retrospective study of forty patients with small (< 20 mm diameter) PD-NEN, including both functional and non-functional tumors, long-acting octreotide was associated with stable disease in 80% of the patients, partial response in 10%, and progressive disease in 10% [66].

Another study compared patients with small (< 20 mm diameter) MEN1-related PNEN receiving long-acting lanreotide to active surveillance. There was a significant difference, as the median time to progression of 91 PNEN was not reached in the treatment arm vs. 40 months in the surveillance group [67]. Another advantage of somatostatin analogs is their ability to ameliorate hypersecretion syndromes, such as in insulinomas [68].

When approaching the issue of preventive medical intervention for small non-functioning DP-NEN, we should consider the substantial evidence of the low risk of these neoplasms and the disadvantages of the life-long treatment required, typically from a young age. Nevertheless, in patients that are at high risk for surgery, had previous surgery, and are at risk for completion pancreatectomy with the abolishment of exocrine and endocrine secretion, or based on the clinician consideration and the discussion with the patients, medical prevention with long-term somatostatin analogs for small DP-NEN is available, well tolerated and safe.

### Pituitary adenomas (PitAd)

The prevalence of PitAd in patients with MEN1 is approximately 40% [69]. Pituitary adenomas are the first clinical manifestation in 13% of patients with MEN1. In patients with MEN1 who develop PitAd during follow-up, it was the presenting manifestation in 31% of patients [69]. Pituitary adenomas are more prevalent in women with MEN1 than in men (46.5% vs. 30.3%, respectively, *p* < 0.001), explained mainly by a higher rate of prolactinomas (25.5% vs. 15.2%, *p* = 0.001), and are also more often the presenting manifestation in women than in men (30.4% vs. 17.7%, *p* < 0.001) [70]. Although most MEN1-related PitAd are microadenomas (diameter < 10 mm), MEN1-related macroadenomas usually entail a more aggressive behavior compared with sporadic PitAd, with visual deficits, suprasellar and cavernous extension as well as reduced rate of remission [71, 72]. In a large cohort of young patients with PitAd, among six children with ACTH-secreting adenomas, five were diagnosed with MEN1, suggesting a higher risk for Cushing’s disease in this population [73].

In a multicenter retrospective study, including 324 patients with MEN1 that were compared with 110 patients with sporadic PitAd, 42% had pituitary adenomas, with a mean age at diagnosis of 38.0 years (range 12–83). PitAd was the presenting manifestation in 17% and was more prevalent among women (50%) than men (31%). In terms of hormone oversecretion, prolactin oversecretion was reported in 62.5%, growth hormone in 8.8%, adrenocorticotrophic hormone (ACTH) in 4.4%, co-secreting (over-secreting more than one hormone) was reported in 9.5% of the adenomas, and 14.7% were non-functioning PitAd. Compared with sporadic PitAd, MEN1-related PitAd were more often macroadenomas (85% vs. only 42% in the sporadic group), and 32% of the MEN1-related PitAd were invasive [72].

Interestingly, despite the relatively weak genotype-phenotype association in MEN1, several variants were identified that were specifically associated with increased risk for PitAd. The *MEN1*-Burin variant [74], detected in a kindred from the Burin peninsula in Canada, is associated with a unique tendency to develop prolactinomas. The *MEN1*-Tasman variant is associated with increased risk for both non-functioning pituitary adenomas and prolactinomas [75].

## Pathogenic variant-negative (“mutation negative”) MEN1

About 10–30% of patients with clinical features of MEN1 are *MEN1*-negative on genetic workup, with the age of clinical presentation usually older than patients with a *MEN1* pathogenic variant [76]. These numbers are possibly an overestimation as they are based on older studies when current genetic sequencing methods were unavailable and before the identification of new MEN1-like syndromes. Several endocrine syndromes mimic MEN1 and should be considered as a possible differential diagnosis in a patient presenting with a suspected MEN1 clinical presentation (e.g., PHPT at a young age, a combination of any two P’s) and no pathogenic variant in the *MEN1* gene. Follow-up in each syndrome is not entirely defined considering the rarity of these syndromes, except for MEN2, and is based on the initial clinical presentation and on expert opinion.

### MEN4 syndrome

Multiple endocrine neoplasia 4 (MEN4) is a relatively recent addition to MEN syndromes, reported in 2006 by Pellegata and colleagues [77]. This autosomal dominant syndrome is caused by a germline loss of function variant in the *CDKN1B* gene, encoding the cell cycle regulator p27 [77]. Interestingly, in sporadic parathyroid adenomas, *MEN1* LOH seems to be associated with reduced expression of p27 [78]. Clinically, patients with MEN4 present mainly with PHPT, PitAd, and PNEN; however, penetrance is lower than that of MEN1 patients. In addition, the age of the PHPT presentation is older, and of PitAd younger than in MEN1. There is a genotype-phenotype correlation, with an increased risk of PHPT in indels compared with point pathogenic variants, with specific locations of variants associated with a greater risk of PHPT [79]. Additional genes of the CDK family should also be considered in similar clinical settings (*CDKN1A*, *CDKN1B*, *CDKN1C*, *CDKN2A*,* CDKN2C*). These genes are weakly associated with features of MEN1-like syndrome. They are not routinely tested in these clinical situations. However, as NGS sequencing is now more readily available, one should consider testing them in highly suspicious clinical cases, and definitely in patients with clinical MEN1 diagnosis but MEN1-negative genetic testing.

### Familial isolated hyperparathyroidism (FIHP)

This autosomal dominant syndrome consists of a PHPT-only phenotype caused by a gain of function germline variant in *GCM2*, a parathyroid development regulator gene [80, 81]. *GCM2*-related FIHP includes an increased rate of multiple gland involvement and recurrence rate compared to sporadic PHPT [82]. In addition, there are several variant hotspots, the most common being p.Tyr394Ser, probably due to a founder effect as it is most prevalent in Ashkenazi Jews [83, 84].

### Familial hypocalciuric hypercalcemia syndrome (FHH)

In this syndrome, patients present with PTH-dependent hypercalcemia and relatively reduced calcium urinary excretion due to a germline variant in the calcium-sensing receptor (*CaSR*) gene or other *CaSR*-related genes (*GNA11*, *AP2S1*). This variant leads to a change in the set-point perception of normal blood calcium levels. Patients with FHH usually have very mild clinical outcomes, if any, and rarely require medical intervention [85]. However, alterations in the *AP2S1* are associated with a more severe phenotype [86].

### Hyperparathyroidism-Jaw tumor syndrome (HPT-JT)

This autosomal dominant disorder is caused by a loss of function pathogenic germline variant in the *CDC73* gene, encoding the parafibromin protein [87]. This syndrome consists of early-onset PHPT, occurring as the first manifestation in over 80% of patients, with a 15% risk of parathyroid carcinoma [88]. Additional clinical manifestations include various potentially malignant jaw, uterine, and kidney pelvic tumors [89].

### MEN2A syndrome

MEN2 is an autosomal dominant disorder caused by a gain of function germline variant in the *RET* gene, in which medullary thyroid carcinoma (MTC) is a hallmark, with almost 100% penetrance. Additional manifestations include pheochromocytoma (in 50% of patients) and PHPT (in up to 35% of patients) [90]. MEN2 syndrome is further subdivided according to phenotype and specific *RET* variant into MEN2A that includes PHPT and MEN2B (also called MEN3) that does not involve PHPT but may include marphanoid appearance and specific skin manifestations. Penetrance and age of MTC development are associated with specific *RET* pathogenic variants, with earlier age of presentation and increased aggressiveness in MEN2B [91]. While there are no known cases of PHPT-only presentation, it is possible, albeit rare (0.9% of cases), that a patient with MEN2 will first present with PHPT, and as part of the workup, an MTC will be diagnosed [92].

### Familial isolated pituitary adenoma (FIPA)

The prevalent cause of FIPA (approximately 20% of cases) is a germline pathogenic variant in the *AIP* gene leading to an autosomal dominant disorder with early age onset of large pituitary adenomas. Some patients may present with pituitary apoplexy as the first clinical manifestation. Most adenomas secrete growth hormone, but other secretory or non-secretory adenomas also occur [93]. Another FIPA-associated syndrome is X-lag, caused by a microduplication within the *GPR101* gene (located on the X chromosome), manifesting with a non-X-linked early-onset pituitary adenomas secreting growth hormone and gigantism [94, 95].

## Advances in translational and clinical research and future perspectives

Recently, we have learned more about the unique characteristics of this unique MEN1 patient population. For example, patients with MEN1 have an increased risk for thromboembolic events [96] and have an increased risk for breast cancer [97] and for urologic [98], soft tissue, skin, and other neoplasms [5], in addition to the main and common manifestations. It should be noted that these data are derived mostly from retrospective studies and should be interpreted cautiously.

Future perspectives in patient surveillance include developing circulating markers for the various manifestations of MEN1. This will delineate the follow-up plan and patient management, especially regarding decision-making on DP-NEN. In a recent international collaborative effort [99], proteomics and gene expression data were co-analyzed, based both on data from MEN1-PNEN mouse model mice Men1^fl/fl^/Pdx1-Cre^Tg^, and plasma samples from 56 patients (14 with advanced DP-NEN, 42 with no/localized DP-NEN). Overall, 19 proteins were associated with DP-NEN progression in patients with MEN1, and if validated, these may be used as circulating markers for advanced DP-NEN.

Identification of the key role of dihydroorotate dehydrogenase (DHODH) in MEN1-related neoplasm development opened a potential new avenue for intervention. DHODH converts dihydroorotate to orotate and is identified as a specific synthetic lethal target of MEN1 deficiency. ​In their translational work, Ma et al. [100] showed that leflunomide, a DHODH inhibitor, efficiently targets MEN1-mutated tumors in cancer cell lines and xenograft models. The drug was initially tested in three patients with disease progression after 1st line treatment. As a next step, the LUMEN1 clinical study will assess the efficacy of leflunomide for patients with genetic MEN1 diagnosis and any tumor or hormone oversecretion syndrome. In this single-arm study initiated in May 2023, fifteen patients are expected to receive leflunomide for six months, and the primary endpoint will be tumor response [101].

A fascinating advance in our understanding of MEN1 arrives from studies showing the role of the *MEN1* gene in other “non-MEN1 syndrome-related” malignancies. These includelymphoma [102], leukemia [103], prostate cancer [104], sarcoma [105] and others. The increasing interest in the potential targetability of menin in multiple prevalent malignancies increases the chance that a targeted therapy will eventually develop for MEN1-related manifestations.

## Data Availability

No datasets were generated or analysed during the current study.
